# Upregulated miR-206 Aggravates Deep Vein Thrombosis by Regulating GJA1-Mediated Autophagy of Endothelial Progenitor Cells

**DOI:** 10.1155/2022/9966306

**Published:** 2022-03-18

**Authors:** Yan Li, Jingping Ge, Yuanyuan Yin, Ruowen Yang, Jie Kong, Jianping Gu

**Affiliations:** Department of Vascular and Interventional Radiology, Nanjing First Hospital, Nanjing Medical University, Nanjing, 210006 Jiangsu, China

## Abstract

**Background:**

Deep vein thrombosis (DVT) is the third most prevalent vascular disease worldwide. MicroRNAs (miRNAs) play regulatory roles in functions of endothelial progenitor cells (EPCs), which is becoming a promising therapeutic choice for thrombus resolution. Nevertheless, the role of miR-206 in EPCs is unclear.

**Methods:**

EPCs were isolated from the peripheral blood of patients with DVT. In DVT mouse models, DVT was induced by stenosis of the inferior vena cava (IVC). The levels of miR-206 and gap junction protein alpha 1 (GJA1) in EPCs and vascular tissues of DVT mice were detected by reverse transcription-quantitative polymerase chain reaction (RT-qPCR). The proliferation, migration, apoptosis, and angiogenesis were tested by cell counting kit-8 (CCK-8) assay, Transwell assay, flow cytometry analysis, and *in vitro* tube formation assay. The levels of autophagy-related proteins as well as the level of GJA1 in EPCs and vascular tissues were evaluated by western blotting. DVT formation *in vivo* was observed through hematoxylin-eosin (HE) staining. The expression of thrombus resolution markers, CD34 molecule (CD34) and matrix metallopeptidase 2 (MMP2), in the thrombi was measured by immunofluorescence staining.

**Results:**

miR-206 overexpression inhibited proliferation, migration, and angiogenesis and promoted apoptosis of EPCs, while miR-206 knockdown exerted an opposite effect on EPC phenotypes. Downregulation of GJA1, the target of miR-206, abolished the influence of miR-206 on EPC phenotypes. Furthermore, silencing of miR-206 suppressed the autophagy of EPCs via upregulating GJA1. miR-206 knockdown repressed thrombus formation, enhanced the homing ability of EPCs to the thrombosis site, and facilitated thrombus resolution in DVT mouse models. Additionally, miR-206 was upregulated while GJA1 was downregulated in vascular tissues of DVT mice. miR-206 knockdown elevated GJA1 expression in vascular tissues of DVT mice. The expression of miR-206 was negatively correlated with that of GJA1 in DVT mice.

**Conclusion:**

miR-206 knockdown upregulates GJA1 to inhibit autophagy of EPCs and then promote EPC proliferation, migration, and angiogenesis, thereby enhancing EPC homing to thrombi and facilitating thrombus resolution.

## 1. Introduction

Deep vein thrombosis (DVT), with an annual incidence of about 10 million people, is the third most prevalent vascular disease worldwide [1]. DVT refers to the formation of blood clots in the deep venous lumen, which leads to blood flow disorders [2]. Clinical symptoms include limb swelling, superficial vein expansion, chronic pain, and distal venous hypertension [3]. At present, effective treatment options for DVT include surgical thrombectomy, pharmacologic thrombolysis, and anticoagulation [4]. Low-molecular-weight heparin (LMWH) is a drug with anticoagulant and antithrombotic effects, which has been widely used in DVT treatment [5]. However, these therapeutic methods also have great side effects, such as wound complications and risk of major hemorrhage [6]. Thus, it is essential to explore the molecular mechanism of DVT to improve its treatment.

Endothelial progenitor cells (EPCs) are vascular endothelial precursor cells, which have been demonstrated to show effective therapeutic applications in cardiovascular- and vascular-related diseases [7]. EPCs have the capacity to proliferate, migrate to peripheral blood, and further differentiate into endothelial cells to participate in angiogenesis [8]. It has been reported that EPCs can be recruited to the thrombus to accelerate thrombus resolution, making EPCs emerge as a promising direction for DVT treatment [9]. However, the clinical applications of EPCs are faced with many challenges [10]. Thus, to explore the mechanism regulating EPC functions is crucial.

MicroRNAs (miRNAs) are small noncoding RNAs of 21-24 nucleotide long, which participate in posttranscriptional modulation of gene expression through suppressing RNA translation or facilitating mRNA degradation [11]. Numerous miRNAs including miR-143-3p, miR-195, and miR-130a-3p play pivotal roles in modulating the functions of EPCs such as cell proliferation, migration, angiogenesis, and autophagy and further influencing DVT recanalization and resolution [12–14]. miR-206 was reported to be implicated in the pathogenesis of many cardiovascular disorders, such as atherosclerosis, pulmonary arterial hypertension, and coronary artery disease (CAD) [15–18]. In a previous study, miR-206 was found to be markedly upregulated in peripheral blood EPCs from patients with CAD, and miR-206 downregulated phosphatidylinositol-4-phosphate 3-kinase catalytic subunit type 2 alpha (PIK3C2*α*) expression to repress the migratory and vasculogenic abilities of EPCs and therefore result in the pathophysiology of CAD [19]. However, whether miR-206 adjusts the functions of EPCs from patients with DVT and thrombus resolution is unclear. Furthermore, it has been widely reported that miR-206 exerts its biological effects in various pathological processes in human diseases via targeting gap junction protein alpha 1 (GJA1), usually known as connexin 43 (Cx43) [20–22]. Gap junctions (GJs) mediate the exchange of metabolites, ions, and secondary messenger molecules between adjacent cells, being a significant contributor to direct cell-to-cell communication [23]. GJs are composed of connexins, and Cx43 has been identified as a major GJ protein in multiple cells, which is crucial for regulating stem cell differentiation [24]. Blocking Cx43 expression has been reported to suppress GJ-mediated signal transfer between neighboring cells, thereby impairing EPC proliferation, migration, and angiogenesis [25]. Therefore, our study investigated whether miR-206 affects EPC proliferation, migration, and angiogenesis in the pathogenesis of DVT via targeting GJA1.

Here, we intended to explore the role and associated molecular mechanism of miR-206 in regulating EPC functions and thrombus resolution. We hypothesized that miR-206 might target GJA1 to mediate the autophagy, proliferation, migration, and angiogenesis of EPCs, thereby regulating thrombus resolution. Our study might provide a new therapeutic target for DVT.

## 2. Materials and Methods

### 2.1. Cell Isolation and Culture

Ten patients aging 18-60 years old, who were diagnosed with first idiopathic DVT of the lower limbs at Nanjing First Hospital, Nanjing Medical University (Jiangsu, China), were included in this study. None of the patients had received anticoagulant drugs or undergone surgery prior to blood collection. DVT were identified by color Doppler ultrasound and lower extremity angiography. Patients with a history of diabetes mellitus, hypertension, and other chronic diseases were excluded. Peripheral blood was collected (80 ml each subject) from ten patients for isolating EPCs. The written informed consents were obtained from all participants. The study was approved by the Ethics Committee of Nanjing First Hospital, Nanjing Medical University (Jiangsu, China).

Human EPCs were extracted as previously described [26]. In brief, mononuclear cells were extracted from the peripheral blood of DVT patients by density-gradient centrifugation. Then, the cells were plated on fibronectin (Corning, NY, USA)-coated T-75 cell culture flask (Thermo Fisher Scientific, Waltham, MA, USA), followed by incubation with endothelial growth medium-2 (Lonza, Walkersville, Maryland, USA) containing 10% fetal bovine serum (FBS; Gibco, Carlsbad, CA, USA). After 4 days of incubation, nonadherent cells were removed by washing with phosphate-buffered saline (PBS) and the medium was changed. Early EPCs developed a spindle-shaped morphology after 7 days of culture. Colonies of endothelial-like cells were grown to 80% confluence, harvested with 0.25% trypsin, and passaged at a ratio of 1 : 2. The third or fourth passages of EPCs were used for the subsequent experiments.

### 2.2. Cell Transfection

Short hairpin RNA targeting GJA1 (sh-GJA1) and negative control (sh-NC) were purchased from RiboBio (Guangzhou, China). miR-206 mimics, miR-206 inhibitor, and the corresponding negative controls (miR-NC) were all synthesized by GenePharma (Shanghai, China). EPCs were inoculated into 6-well plates (2 × 10^5^ cells/well), and transfection was performed using Lipofectamine 2000 (Invitrogen, CA, USA) when confluence reached 50%. After 48 h, the transfection efficiency was detected by reverse transcription-quantitative polymerase chain reaction (RT-qPCR).

### 2.3. DVT Mouse Model Establishment

Eight-week-old male C57BL/6J mice were purchased from Beijing Vital River Laboratory Animal Technology Co. Ltd. Animal experiment was approved by the Institutional Animal Care and Use Committee of Nanjing First Hospital, Nanjing Medical University (Jiangsu, China).

The inferior vena cava (IVC) stenosis-induced DVT mouse model was established as described previously [27]. Briefly, mice were anesthetized with isoflurane-oxygen mixture. An incision was made along the midline of the abdomen, and intestines were exteriorized and soaked in warm saline. The IVC was gently separated from the aorta, and all IVC side branches were ligated. Subsequently, a 7.0 polypropylene suture was placed over the IVC and ligated over a spacer (30-gauge needle), and the spacer was then removed. This procedure led to about 90% closure of the vessel lumen without endothelial denudation. Finally, the peritoneum was closed with silk suture and skin with staples. After 24 h, thrombus formation in the IVC was checked.

### 2.4. Animal Grouping for Detecting Thrombus Formation in DVT Mice

Forty mice were allocated into 4 groups (10/group) as follows: (1) Sham group (mice received the same surgical procedure above without IVC-stenosis), (2) DVT group (mice underwent the above IVC-stenosis operation after injection with 200 *μ*l saline via tail vein), (3) DVT+antagomir-NC group, and (4) DVT+antagomir-206 group. Ten nmol antagomir-NC or antagomir-206 (RiboBio) in 200 *μ*l of saline were injected into the tail vein of each mouse. The above treatments were performed 30 min before the surgery. Twenty-four h after the operation, mice were sacrificed [28].

### 2.5. Animal Grouping for Evaluating Thrombus Recanalization and Resolution in DVT Mice

Another twenty mice were chosen to undergo the same IVC-stenosis operation as above to establish DVT mouse models. Forty-eight h after the establishment of DVT mouse models, twenty mice were injected with 5 × 10^6^ GFP-NC inhibitor-EPCs (*n* = 10) or GFP-miR-206 inhibitor-EPCs (*n* = 10) via the tail vein. After 7 days, thrombi with vessel walls were harvested for measuring size and weight. To analyze the homing ability of EPCs, an IX-81 laser confocal microscope (Olympus) was used to observe the number of GFP-EPCs at the thrombotic site.

### 2.6. Immunofluorescence Staining

To immunostain MMP2 and CD34, frozen sections of thrombus were stained with anti-MMP2 (ab92536; 1 : 250; Abcam, Cambridge, UK) and anti-CD34 (ab81289; 1 : 200; Abcam). After 1 h incubation at room temperature with fluorescent-labeled secondary antibodies (goat anti-mouse Alexa Fluor® 647; ab150115; 1 : 1000; Abcam), a confocal microscope was used to capture the images under the same conditions for each experiment.

### 2.7. RNA Extraction and RT-qPCR

Total RNAs were extracted from EPCs and mouse vascular tissues using Trizol (Invitrogen). The isolation of miRNAs was implemented using the mirVana miRNA Isolation Kit (Invitrogen). The PrimeScript RT reagent kit (TaKaRa, Dalian, China) or PrimeScript miRNA cDNA Synthesis Kit (TaKaRa) was employed for reverse transcription of mRNAs or miRNAs, respectively. RT-qPCR was performed using the SYBR Green qPCR Master Mix Kit (Thermo Fisher Scientific) on the ABI 7500 Fast Real-Time PCR System (Applied Biosystems, Thermo Fisher Scientific). U6 and GAPDH were used as internal controls. The relative gene expression was calculated using the 2^-*ΔΔ*Ct^ method. All primers are listed in Table 1.

### 2.8. Cell Counting Kit-8 (CCK-8) Assay

The proliferation of EPCs was evaluated using the CCK-8 kit (Dojindo, Kumamoto, Japan). In brief, 1 × 10^4^ cells with 100 *μ*l of EGM-2 were inoculated into each well of 96-well plates, followed by 24 h of incubation. Then, each well was added with a new medium containing CCK-8 reagent (*v*/*v* = 10 : 1), and cells were further cultured for 2 h at 37°C. The optical density (OD) value at 450 nm was examined using a microplate reader (Thermo Fisher Scientific).

### 2.9. Transwell Migration Assay

The 24-well Transwell chambers (8 *μ*m pore size; Corning) were used to evaluate cell migration. Transfected EPCs (5 × 10^4^) were resuspended in 250 *μ*l of serum-free EBM-2 medium and were added to the upper chamber. Then, 600 *μ*l of EGM-2MV medium containing 10% FBS was added to the lower chamber. After 24 h of incubation, residual cells in the upper chamber were scraped off using a cotton swab. The migrated cells in the lower chamber were stained with 0.1% crystal violet (Sigma-Aldrich, St. Louis, MO, USA). Images of the stained cells from 5 random fields were captured using a microscope (Leica, Germany).

### 2.10. Flow Cytometry Analysis

Cell apoptosis was measured applying the PI/Annexin V stain (Sigma-Aldrich) method. Transfected EPCs were digested with 0.25% trypsin solution and washed with cold PBS. Then, cells (1 × 10^5^ cells/ml) were stained with binding buffer containing 10 *μ*l of Annexin V-FITC and 5 *μ*l of propidine iodide (PI) at 4°C for 15 min in the dark. Subsequently, cells were analyzed using flow cytometry (Beckman FC400 MPL, USA).

### 2.11. Tube Formation Assay

The angiogenic capacity of EPCs was detected through *in vitro* tube formation assay. Approximately 40 *μ*l of Matrigel (R&D Systems, MN, USA) was added into each well of 96-well plates, followed by 30 min of culture for solidification. Then, 100 *μ*l of EPCs was inoculated into the coated plates. After culturing at 37°C for 8 h, capillary-like structures could be observed under a microscope.

### 2.12. Luciferase Reporter Assay

The binding site of miR-206 on GJA1 3′UTR is predicted at the TargetScan database (http://www.targetscan.org/). The sequences of GJA1 3′UTR containing the potential wild-type (Wt) or mutant (Mut) binding sites of miR-206 were inserted into the pmiR-RB-REPORT vector (RiboBio) to construct the firefly luciferase reporter plasmids. Then, pmiR-RB-REPORT-GJA1-3′UTR-Wt or pmiR-RB-REPORT-GJA1-3′UTR-Mut was cotransfected with miR-NC or miR-206 inhibitor into EPCs using Lipofectamine 2000. After 48 h, the activities of luciferase and Renilla plasmid were detected by the Dual-Luciferase Reporter 1000 Assay System (Promega).

### 2.13. Western Blotting

EPCs and vascular tissues were lysed using radio-immunoprecipitation assay (RIPA) buffer (Sigma-Aldrich) containing protease inhibitors. The concentration of proteins was measured using the bicinchoninic acid (BCA) kit (Sigma-Aldrich). Then, protein samples (30 *μ*g) were electroblotted onto polyvinylidene fluoride (PVDF) membranes (Millipore) after resolving in 12% sodium dodecyl sulfate-polyacrylamide gel electrophoresis (SDS-PAGE). The membranes were blocked with 5% bovine serum albumin (BSA), followed by incubation with primary antibodies against GJA1 (ab230537, 1 : 1000, Abcam), histone deacetylase 3 (HDAC3; ab32369, 1 : 5000, Abcam), monocyte to macrophage differentiation associated (MMD; ab173967, 1 : 500, Abcam), solute carrier family 44 member 1 (SLC44A1; ab110767, 1 : 500, Abcam), paired box 7 (PAX7; ab187339, 1 : 1000, Abcam), cyclin-dependent kinase 14 (CDK14; #43794, 1 : 200, Signalway Antibody, Greenbelt, Maryland, USA), cadherin like and PC-esterase domain containing 1 (CPED1; ab181051, 1 : 1000, Abcam), small integral membrane protein 14 (SMIM14; ab204555, 0.1 *μ*g/ml, Abcam), transgelin 2 (TAGLN2; ab121146, 0.1 *μ*g/ml, Abcam), tankyrase 2 (TNKS2; ab155545, 1 : 500, Abcam), autophagy-related 5 (ATG5; ab108327, 1 : 1000), autophagy-related 7 (ATG7; ab133528, 1 : 10000), LC3 I/II (ab51520, 1 : 3000), p62 (ab240635, 1 : 1000), and *β*-actin (ab8227, 1 : 1000) overnight at 4°C. The membranes were further incubated with horseradish peroxidase-conjugated goat anti-rabbit immunoglobulin G (IgG) antibody (ab97051, 1 : 2000; Abcam) at 37°C for 1 h. Band intensities were determined using ImageJ software (NIH, Bethesda, MD, USA).

### 2.14. Hematoxylin and Eosin (HE) Staining

After the mice were sacrificed, sections of the specimens 2 mm below the IVC ligation were removed and fixed with 4% paraformaldehyde for histological analysis. The fixed specimens were dehydrated using gradient alcohol, cleared up with xylene, and embedded in paraffin. The embedded tissue blocks were sliced at 4 *μ*m intervals. These sections were later stained with hematoxylin for 15 min, washed with water, counterstained with eosin for 5 min, dehydrated with alcohol, hyalinized, and mounted with neutral resins. Finally, an inverted microscope (Olympus, Tokyo, Japan) was used to capture all histological images.

### 2.15. Statistical Analysis

Experimental data from at least three independent experiments were analyzed using SPSS 20.0 (SPSS, Chicago, USA) and are presented as the mean ± standard deviation. Student's *t*-test or one-way analysis of variance (ANOVA) followed by the Tukey *post hoc* test was employed to compare differences between groups or among groups. Gene correlation was analyzed by Pearson correlation analysis. In all analyses, *p* < 0.05 was considered significant.

## 3. Results

### 3.1. miR-206 Affects EPC Proliferation, Apoptosis, Migration, and Angiogenesis

**Figure 1:**
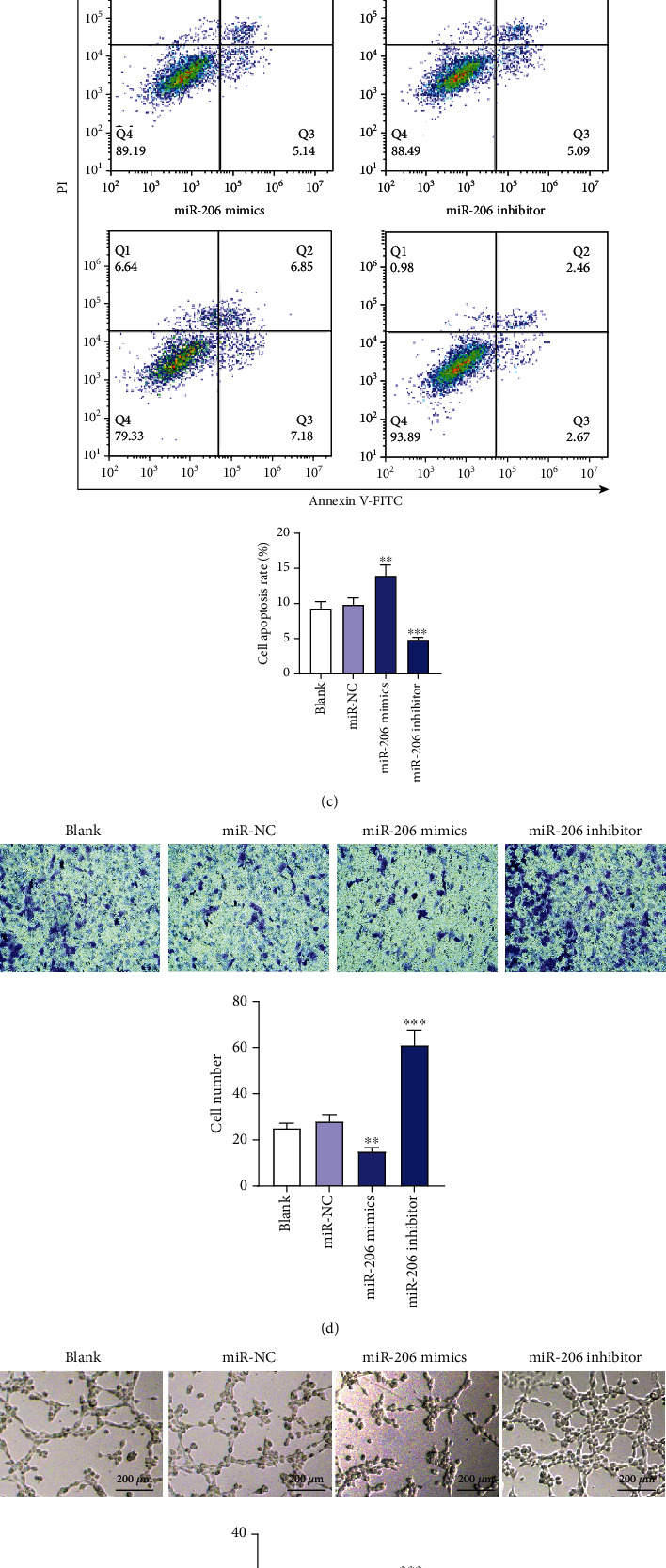
The influence of miR-206 on EPC proliferation, apoptosis, migration, and angiogenesis. (a) PCR analysis of miR-206 expression in EPCs after transfection with miR-NC, miR-206 mimics, or miR-206 inhibitor. (b) CCK-8 assay of EPC proliferation after the above transfection. (c) Flow cytometry analysis of the apoptosis of EPCs after the above transfection. (d) Transwell assay of the migration of EPCs after the above transfection. Representative images of cell migration and the number of migratory cells are shown. (e) *In vitro* tube formation assay of the angiogenesis of EPCs after the above transfection. Representative images of capillary tube formation and the number of tubes are shown. ^∗∗^*p* < 0.01 and ^∗∗∗^*p* < 0.001.

To investigate the influence of miR-206 on the phenotypes of EPCs, we first transfected EPCs with miR-206 inhibitor or mimics. PCR analysis indicated that miR-206 expression in EPCs was reduced after miR-206 downregulation but was elevated after miR-206 overexpression (Figure 1(a)). Then, a series of loss-of-function or gain-of-function experiments were conducted. As shown by the CCK-8 assay, miR-206 downregulation facilitated while miR-206 overexpression inhibited the EPC proliferation (Figure 1(b)). The results of flow cytometry analysis demonstrated that the percentage of apoptotic cells in the miR-206 inhibitor group was decreased, while the apoptotic cell population in the miR-206 mimic group was increased (Figure 1(c)). Transwell migration assay was conducted to evaluate EPC migration, which demonstrated that the number of EPCs passing through the membrane into the lower chamber was increased after miR-206 knockdown, while this number was significantly reduced after overexpressing miR-206 (Figure 1(d)). Additionally, to investigate whether miR-206 affects the angiogenesis of EPCs, a tube formation assay was carried out. Compared with the miR-NC group, EPCs transfected with miR-206 inhibitor displayed increased capillary-like structures and tube number, while EPCs transfected with miR-206 mimics showed decreased capillary-like structures and tube number (Figure 1(e)). This suggested that miR-206 exerted a negative effect on EPC angiogenesis. In conclusion, miR-206 knockdown promotes the proliferation, migration, and angiogenesis as well as inhibits the apoptosis of EPCs, while miR-206 overexpression exerts the opposite effects.

### 3.2. miR-206 Directly Targets GJA1

**Figure 2:**
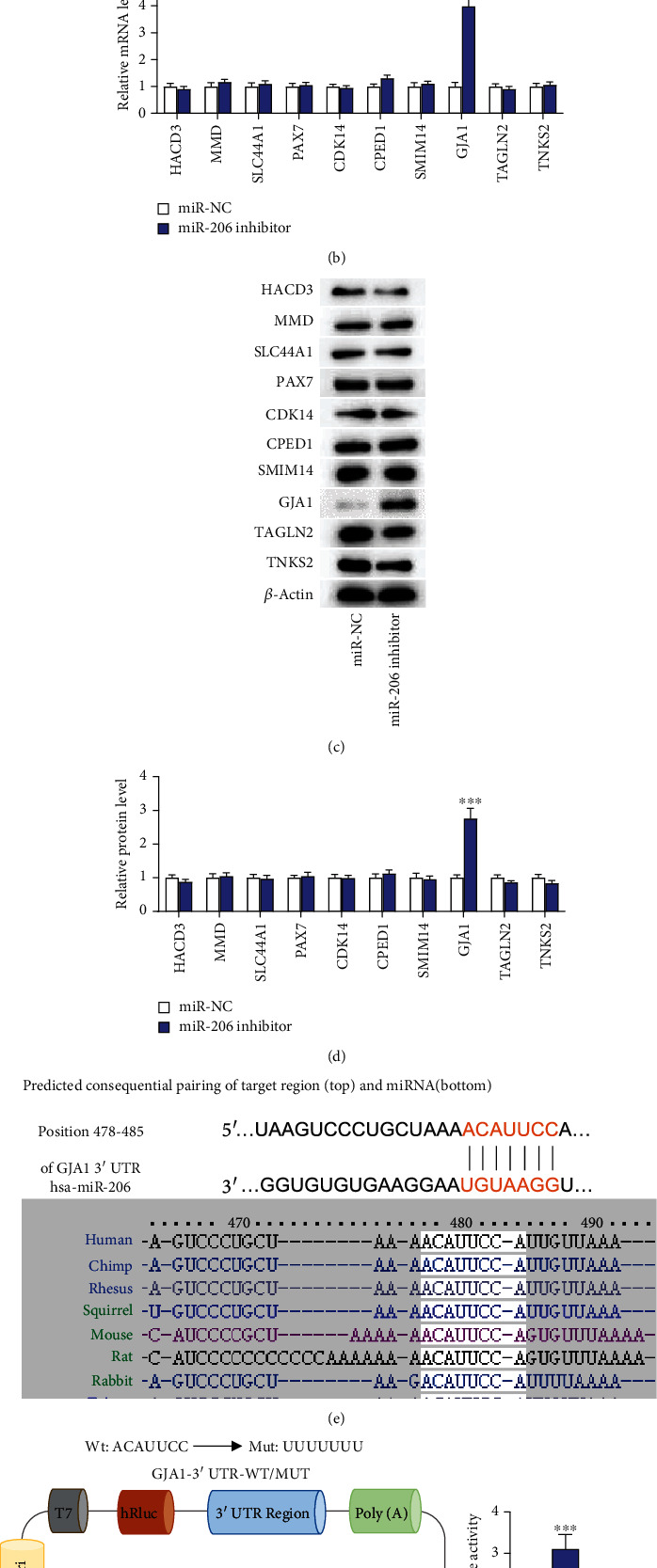
miR-206 directly targets GJA1. (a) The downstream targets of miR-206 were predicted at the miRDB database, and the top ten were selected as candidates. (b) PCR analysis of the expression of the above ten candidate targets in EPCs after downregulating miR-206. (c, d) Western blotting analysis of the protein levels of ten candidate targets in EPCs after knocking down miR-206. (e) The TargetScan database revealed the binding site of miR-206 on GJA1 3′UTR. (f, g) Luciferase reporter assay of the luciferase activities of pmiR-RB-REPORT-GJA1-3′UTR-Wt/Mut vectors in EPCs after miR-206 downregulation. ^∗∗∗^*p* < 0.001.

Since the influence of miR-206 on EPC phenotypes was confirmed, we subsequently investigated the underlying mechanism by which miR-206 regulates the behavior of EPCs. Based on the miRDB database (http://mirdb.org/), the target genes of miR-206 were predicted and the top ten genes were selected for PCR and western blotting analysis (Figure 2(a)). We discovered that versus the miR-NC group, miR-206 knockdown affected the mRNA and protein levels of ten genes in EPCs, among which GJA1 displayed the most significant change. The mRNA and protein levels of GJA1 in EPCs were markedly elevated after downregulating miR-206 (Figures 2(b)–2(d)). Therefore, miR-206 negatively regulated the expression of GJA1 in EPCs. The TargetScan database revealed the binding sequence of miR-206 on position 478-485 of GJA1 3′UTR, and the binding site is highly conserved among multiple species (Figure 2(e)). To further validate whether miR-206 binds to the GJA1 3′UTR, a luciferase reporter assay was conducted. Wild-type or mutant 3′UTR of GJA1 containing the miR-206 binding site were cloned into pmiR-RB-REPORT luciferase reporter vector for recombinant vectors pmiR-RB-REPORT-GJA1-3′UTR-Wt/Mut (Figure 2(f)). miR-206 knockdown remarkably enhanced the luciferase activity of pmiR-RB-REPORT-GJA1-3′UTR-Wt, while there was no significant change in the luciferase activity of pmiR-RB-REPORT-GJA1-3′UTR-Mut (Figure 2(g)). Overall, GJA1 is a target of miR-206.

GJA1 silencing attenuates the influence of miR-206 downregulation on EPC proliferation, apoptosis, migration, and angiogenesis.

**Figure 3:**
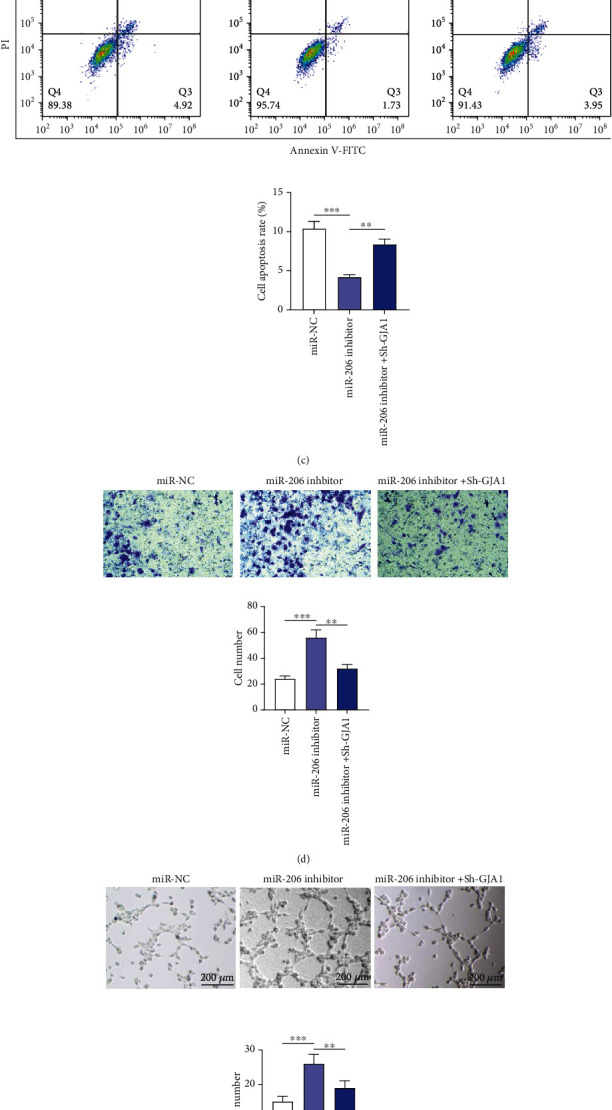
GJA1 silencing attenuates the influence of miR-206 downregulation on EPC proliferation, apoptosis, migration, and angiogenesis. (a) Western blotting analysis of GJA1 protein level in EPCs after GJA1 silencing. (b) CCK-8 assay of EPC proliferation at 0, 24, 48, 72, and 96 h after miR-NC, miR-206 inhibitor, or miR-206 inhibitor+sh-GJA1 transfection. (c) Flow cytometry analysis of the apoptosis of EPCs after the above transfection. (d) Transwell migration assay of cell migration at 24 h after the above transfection. (e) *In vitro* tube formation assay of EPC angiogenesis after the above transfection. Representative images of capillary tube formation and the number of tubes are shown. ^∗^*p* < 0.05, ^∗∗^*p* < 0.01, and ^∗∗∗^*p* < 0.001.

To further explore whether miR-206 regulates the biological behaviors of EPCs by targeting GJA1, GJA1 was knocked down using sh-GJA1. GJA1 protein level was reduced in EPCs after GJA1 silencing (Figure 3(a)). Subsequently, EPCs were cotransfected with sh-GJA1 and miR-206 inhibitor. We discovered that compared with the miR-NC group, the miR-206 inhibitor group displayed enhanced cell proliferation, migration, and angiogenesis and decreased cell apoptosis (Figures 3(b)–3(e)). However, compared to the miR-206 inhibitor group, the miR-206 inhibitor+sh-GJA1 group showed decreased cell proliferation, migration, and angiogenesis and enhanced cell apoptosis (Figures 3(b)–3(e)). Therefore, cotransfection of miR-206 inhibitor and sh-GJA1 in EPCs effectively antagonized the effects of miR-206 inhibitor alone on EPC behaviors, which suggests that miR-206 regulates EPC behaviors by targeting GJA1.

### 3.3. miR-206 Modulates the Autophagy of EPCs via Targeting GJA1

**Figure 4:**
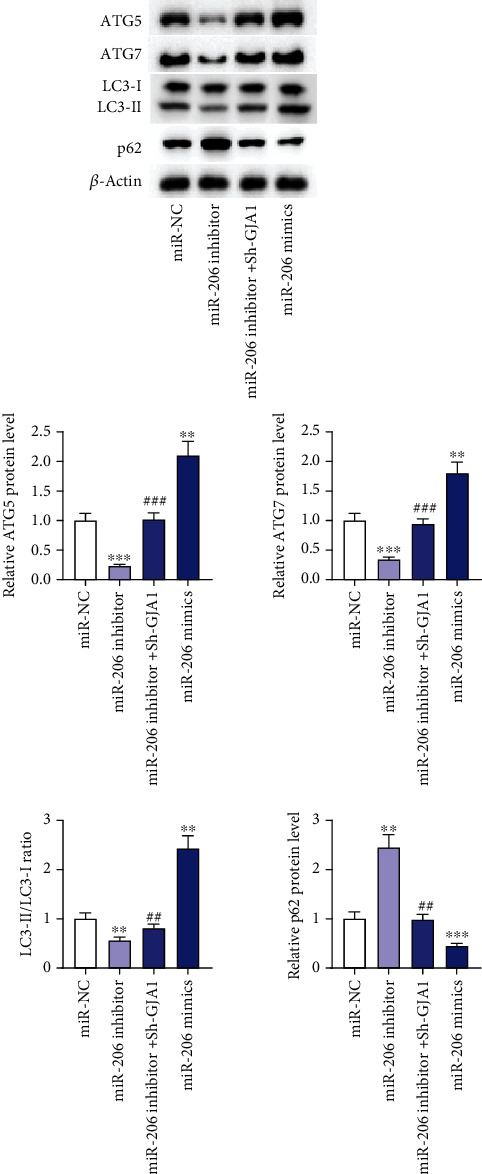
miR-206 modulates the autophagy of EPCs via targeting GJA1. (a) Western blotting analysis of levels of autophagy-related proteins in EPCs after miR-NC, miR-206 inhibitor, miR-206 inhibitor+sh-GJA1, or miR-206 mimic transfection. ^∗∗^*p* < 0.01 and ^∗∗∗^*p* < 0.001 versus miR-NC; ^##^*p* < 0.01 and ^###^*p* < 0.001 versus miR-206 inhibitor

Autophagy is an important metabolic way, and existing studies have demonstrated that autophagy can regulate numerous cell biological activities, including cell growth, apoptosis, migration, and angiogenesis [29]. Since we have confirmed the influence of miR-206 on EPC biological behaviors, then we investigated whether miR-206 regulates autophagy of EPCs by detecting levels of autophagy-related proteins. Western blotting analysis revealed that miR-206 knockdown significantly decreased the protein levels of ATG5 and ATG7 and the ratio of LC3B II/I as well as increased the protein level of p62, while miR-206 overexpression showed the opposite results (Figure 4(a)). These findings indicated that miR-206 knockdown repressed while miR-206 overexpression promoted the autophagy of EPCs. However, we also discovered that GJA1 silencing reversed the influence of miR-206 downregulation on the level of these proteins, which suggested that GJA1 silencing decreased the inhibition of miR-206 downregulation on the EPC autophagy. In summary, miR-206 modulates the autophagy of EPCs via targeting GJA1.

### 3.4. miR-206 Knockdown Represses Thrombus Formation in Mice with DVT

**Figure 5:**
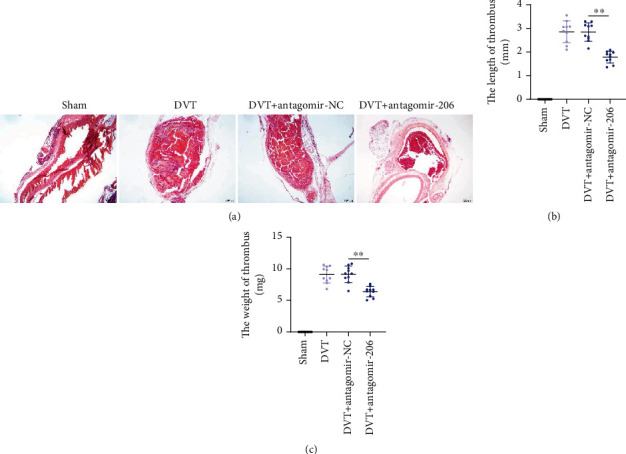
miR-206 knockdown alleviates thrombus formation in mice with DVT. (a) The pathological changes were observed through the HE staining of serial cross sections of IVC at 24 h postoperation from mice in Sham, DVT, DVT+antagomir-NC, or DVT+antagomir-206 groups. (b, c) The length and weight of thrombus at 24 h postoperation from mice in the above four groups. *n* = 10 in each group. ^∗∗^*p* < 0.01.

Since the influence of miR-206 on EPC biological behaviors *in vitro* has been confirmed, then we next established the DVT mouse model to detect whether miR-206 affects the thrombosis in IVC *in vivo*. Through HE staining, we discovered that there was no thrombosis in IVC of mice in the Sham group. Compared with the Sham group, mice in the DVT group displayed obvious thrombosis in IVC 24 h after IVC ligation (Figure 5(a)). However, after injection with antagomir-206, DVT mice showed alleviated blood clots in IVC (Figure 5(a)). In addition, both thrombus length and weight were significantly increased in the DVT group versus the Sham group but were markedly decreased in the DVT+antagomir-206 group versus the DVT+antagomir-NC group (Figures 5(b) and 5(c)). This suggested that miR-206 knockdown reduced the length and weight of the thrombus. Overall, miR-206 knockdown repressed thrombus formation in DVT mouse models.

### 3.5. miR-206 Knockdown Enhances EPC Homing Ability and Facilitates Thrombus Recanalization and Resolution in Mice with DVT

**Figure 6:**
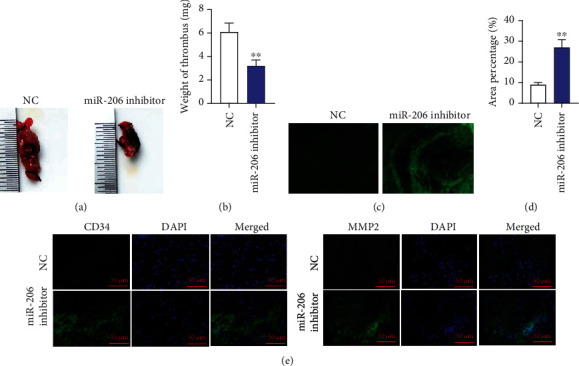
miR-206 knockdown enhances EPC homing ability and facilitates thrombus recanalization and resolution in mice with DVT. (a, b) DVT mouse models were injected with GFP-NC inhibitor-EPCs or GFP-miR-206 inhibitor-EPCs. Size and weight of thrombus at day 7 after the injection. (c, d) The homing ability of EPCs to the thrombosis site at day 7 after the injection was evaluated by calculating the percentage of green-fluorescent area. (e) The expression of CD34 (red) and MMP2 (red) in thrombus was detected by immunofluorescence. *n* = 10 in each group. ^∗∗^*p* < 0.01.

It was reported that EPCs can be recruited to the thrombosis site and contribute to neovascularization and thrombus resolution [30]. Then, we explored whether miR-206 knockdown affects EPC homing ability to the thrombosis site in IVC of DVT mouse models and participates in thrombus recanalization and resolution. EPCs transfected with NC inhibitor or miR-206 inhibitor were injected into DVT mouse models. After 7 days, the thrombi with vessel walls were harvested. The thrombus size in the GFP-miR-206 inhibitor-EPC group was smaller than that in the GFP-NC inhibitor-EPCs group (Figure 6(a)). The thrombus weight was also significantly reduced after injection with GFP-miR-206 inhibitor-EPCs (Figure 6(b)). Furthermore, we discovered that the number of EPCs in the thrombosis site in the GFP-miR-206 inhibitor-EPC group was more than that in the GFP-NC inhibitor-EPCs group (Figures 6(c) and 6(d)). Additionally, immunofluorescence staining was performed to evaluate the expression of CD34, the marker of angiogenesis in the thrombus, and MMP2, a crucial modulator of early thrombus resolution, in the thrombi. The results showed that CD34 and MMP2 displayed higher expression in the thrombi of the GFP-miR-206 inhibitor-EPC group versus the GFP-NC inhibitor-EPC group (Figure 6(e)). In conclusion, miR-206 knockdown enhances EPC homing ability, thereby accelerating thrombus recanalization and resolution.

### 3.6. miR-206 Negatively Regulates GJA1 Expression *In Vivo*

**Figure 7:**
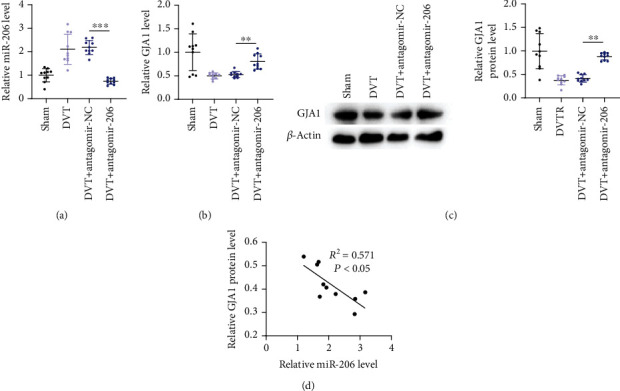
miR-206 negatively regulates GJA1 expression *in vivo.* (a, b) PCR analysis of miR-206 and GJA1 expression in vascular tissues of mice in Sham, DVT, DVT+antagomir-NC, or DVT+antagomir-206 groups (*n* = 10 in each group) was assessed by RT-qPCR. (c) Western blotting analysis of GJA1 protein level in the above four groups (*n* = 10 in each group). (d) Pearson correlation analysis of the correlation between miR-206 and GJA1 in vascular tissues of DVT mice (*n* = 30). ^∗∗^*p* < 0.01 and ^∗∗∗^*p* < 0.001.

Finally, we investigated the expression of miR-206 and GJA1 in vascular tissues of DVT mice. PCR analysis revealed that miR-206 was upregulated while GJA1 was downregulated in vascular tissues of mice in the DVT group. The results of PCR in the DVT+NC group showed no significant difference from those in the DVT group (Figures 7(a) and 7(b)). However, miR-206 expression was reduced, while GJA1 expression was elevated after downregulating miR-206 (Figures 7(a) and 7(b)). Furthermore, the protein level of GJA1 in vascular tissues was also reduced in the DVT group versus the Sham group but was increased in the DVT+antagomir-206 group (Figure 7(d)). Additionally, Pearson correlation analysis demonstrated that miR-206 expression was negatively correlated to GJA1 expression in vascular tissues of DVT mice (Figure 7(d)). In summary, miR-206 negatively regulates GJA1 expression *in vivo.*

## 4. Discussion

DVT is one common venous thromboembolic disorder and will result in deadly pulmonary embolisms if undetected and untreated timely [1]. The existing treatments might also bring unsatisfactory side effects despite their effective therapeutic outcomes [1]. Increasing studies have demonstrated the significant role of EPCs in thrombus resolution and recanalization [4]. miRNAs exert significant effects on the angiogenic capacity of EPCs, which subsequently mediate thrombus recanalization [31]. In this study, we discovered that miR-206 downregulation promotes EPC autophagy, proliferation, migration, and angiogenesis and thereby enhances thrombus resolution by targeting GJA1.

Accumulating studies have shown that miRNAs regulate diverse biological processes, including cell differentiation, apoptosis, survival, and proliferation [32]. miR-206 was previously reported to regulate cellular processes of EPCs, whose angiogenic ability is closely related to the pathology of cardiovascular diseases [33]. For example, miR-206 represses the viability and migration and enhances the apoptosis of EPCs from patients with CAD through inhibiting vascular endothelial growth factor (VEGF) expression, which suggests that miR-206 can be a novel biomarker for CAD [16]. Furthermore, miR-206 inhibits EPC migration, tube formation, and angiogenesis of patients with CAD [19]. In this study, we similarly discovered that miR-206 repressed the growth, migration, and angiogenesis of EPCs from patients with DVT.

GJA1, also known as Cx43, is a significant gap junction protein that acts as a communication channel linking cardiomyocytes [34]. Cx43 is closely correlated with the enhanced migration of several cell types such as glioma cells, or neural crest cells, HeLa cells, and EPCs [35]. By far, Cx43 is the dominant gap junction protein in the EPCs [25]. It was reported that blocking GJA1 expression impairs EPC proliferation, migration, and angiogenesis [35]. In a previous study, VEGF induces EPC differentiation via Cx43-induced GJs and then contributes to vascular repair [36]. In hind limb ischemia mice, GJA1 downregulation reduces VEGF expression and attenuates the proliferation and migration activity as well as the angiogenic potential of EPCs [25]. In this study, we discovered that GJA1 silencing reversed the influence of miR-206 silencing on EPC biological behaviors. GJA1 knockdown promoted the apoptosis while inhibiting the growth, migration, and angiogenesis of EPCs. Therefore, miR-206 contributes to EPC dysfunction by downregulating GJA1.

GJA1 was reported to maintain cell junction structure and cell homeostasis via modulating autophagy [37]. Autophagy is a catabolic process for the degradation and recycling of cytosolic components by lysosomes [38]. Autophagy not only participates in cellular development and homeostasis but also plays a dual role in various physiological processes [39]. Previous studies have demonstrated that autophagy could either negatively or positively regulate cell proliferation, migration, and angiogenesis [40]. For example, miR-143-3p targets autophagy-related 2B (ATG2B) to suppress autophagy and thereby promotes EPC viability, migration, and tube formation [14]. miR-195 inhibits autophagy and thereby attenuates EPC proliferation, migration, and angiogenesis via targeting GABA type A receptor-associated protein-like 1 (GABARAPL1) [12]. In this study, miR-206 knockdown markedly reduced the expression of ATG5 and ATG7 and the ratio of LC3B II/I as well as increased the expression of the ratio of p62, while miR-206 overexpression brought the opposite results. Overall, miR-206 knockdown inhibited while miR-206 overexpression promoted the autophagy of EPCs. However, GJA1 downregulation reversed the effects of miR-206 knockdown on autophagy. Therefore, our study indicated that miR-206 targeted GJA1 to affect autophagy, thereby modulating EPC proliferation, apoptosis, migration, and angiogenesis.

Since the enhanced proliferation, migration, and angiogenesis of EPCs contributes to thrombus resolution, which alleviates the development of DVT, then DVT mouse models were established for investigating the influence of miR-206 on DVT formation and resolution *in vivo*, respectively. We discovered that miR-206 downregulation not only inhibited thrombus formation but also enhanced EPC homing to the thrombosis site and promoted thrombus resolution. This result is in consistent with that in the previous studies [26, 41, 42]. Additionally, upregulated miR-206 and downregulated GJA1 along with a negative correlation between their expression were discovered in vascular tissues of DVT mice, which also demonstrated that miR-206 inhibited GJA1 expression *in vivo*.

## 5. Conclusion

In summary, this study elucidated that miR-206 knockdown targets GJA1 to inhibit EPC autophagy and thereby promote EPC proliferation, migration, and angiogenesis as well as suppress EPC apoptosis, which eventually enhances thrombus resolution and alleviates DVT development. This research might provide a promising target for DVT treatment.

## Figures and Tables

**Table 1:** Primer sequences for RT-qPCR.

Gene	Primer sequences
miR-206	Forward: 5′-CGTCAGAAGGAATGATGCACAG-3′
	Reverse: 5′-ACCTGCGTAGGTAGTTTCATGT-3′
U6	Forward: 5′-CTCGCTTCGGCAGCACA-3′
	Reverse: 5′-AACGCTTCACGAATTTGCGT-3′
HACD3	Forward: 5′-CTCTGATCCAGCTTCTTGGA-3′
	Reverse: 5′-AACCACAGCTTTGTTCTGC-3′
MMD	Forward: 5′-ATGAACCATCGAGCTCCAG-3′
	Reverse: 5′-ACAATGAGGAATGCGTGTG-3′
SLC44A1	Forward: 5′-TGGAAATATCTGTGGGCAG-3′
	Reverse: 5′-AATACATACTTCCGCTGGG-3′
PAX7	Forward: 5′-ACTACCCAGACATATACACCC-3′
	Reverse: 5′-TACTGAACCAGACCTGCAC-3′
CDK14	Forward: 5′-CAGATTTCGGTCTTGCAAGAG-3′
	Reverse: 5′-GGAGGTCTGTACCACAAGG-3′
CPED1	Forward: 5′-CAAAGACCTTCCATAGATGCAG-3′
	Reverse: 5′-CTACAGGGTACATGAAACTGC-3′
SMIM14	Forward: 5′-TCTTGTTCTTACTGAGACCTCC-3′
	Reverse: 5′-GATCTTGTCCATTATGAGGACTG-3′
GJA1	Forward: 5′-TTCAATCACTTGGCGTGAC-3′
	Reverse: 5′-CAAGGAGTTTGCCTAAGGC-3′
TAGLN2	Forward: 5′-CTGGGAAGGAAAGAACATGG-3′
	Reverse: 5′-AGAAGAGCCCATCATCTCG-3′
TNKS2	Forward: 5′-TGGCAGAAAGTCAACTCCA-3′
	Reverse: 5′-GCTCCATGTTGCAGTAACAG-3′
GAPDH	Forward: 5′-GCATCCTGGGCTACACTG-3′
	Reverse: 5′-TGGTCGTTGAGGGCAAT-3′

## Data Availability

The datasets used during the current study are available from the corresponding author on reasonable request.
